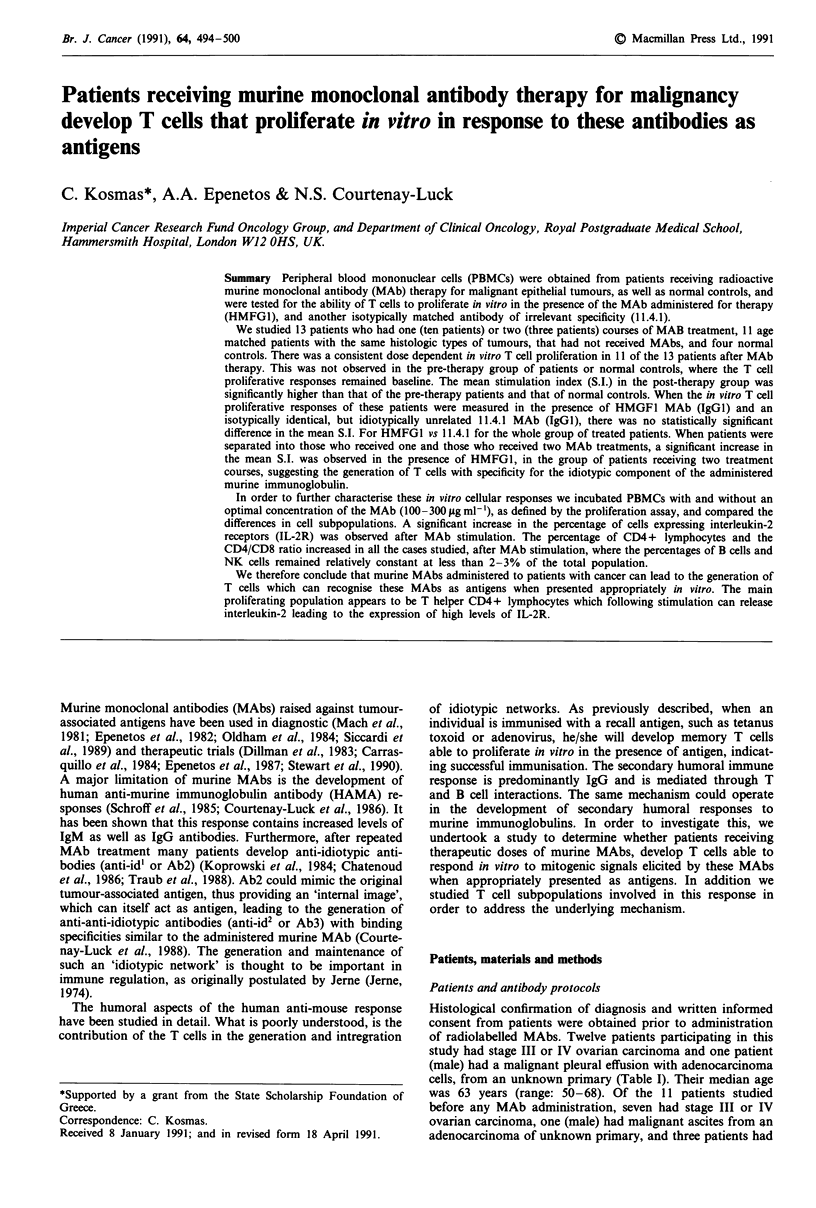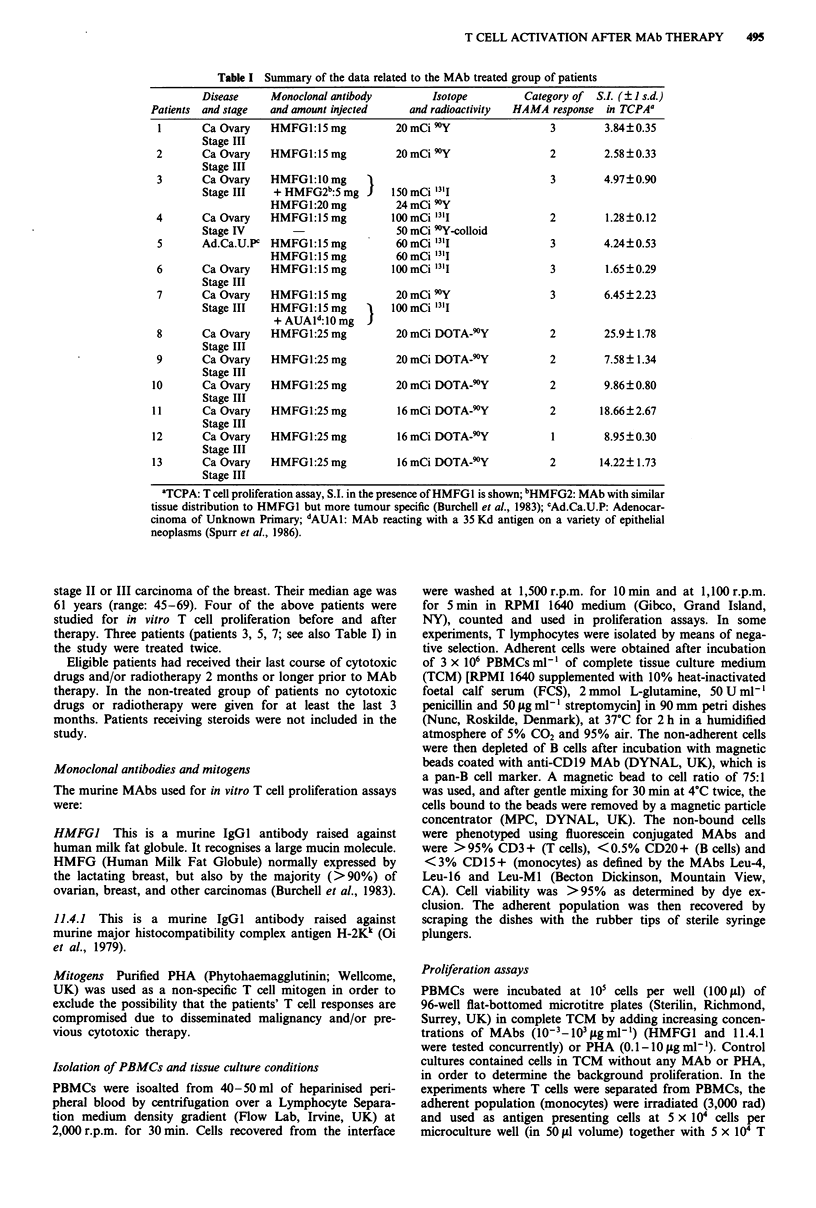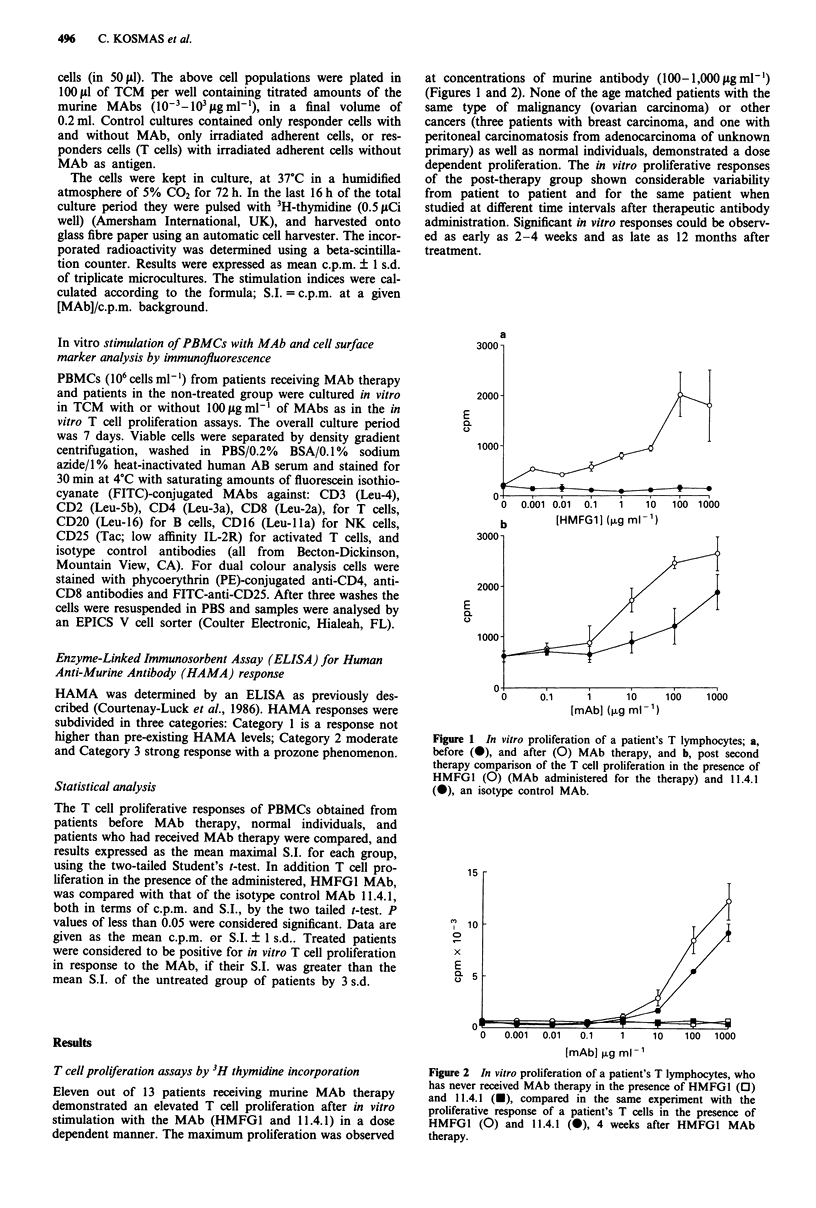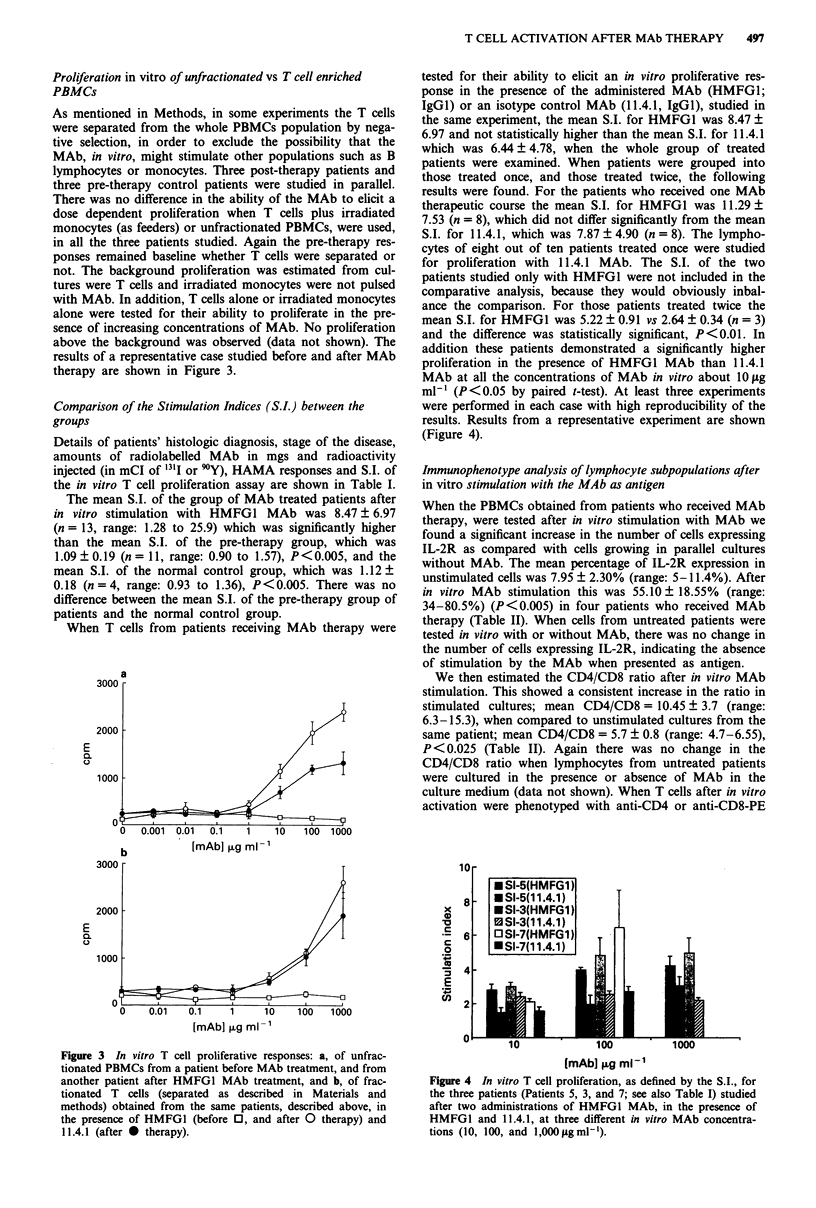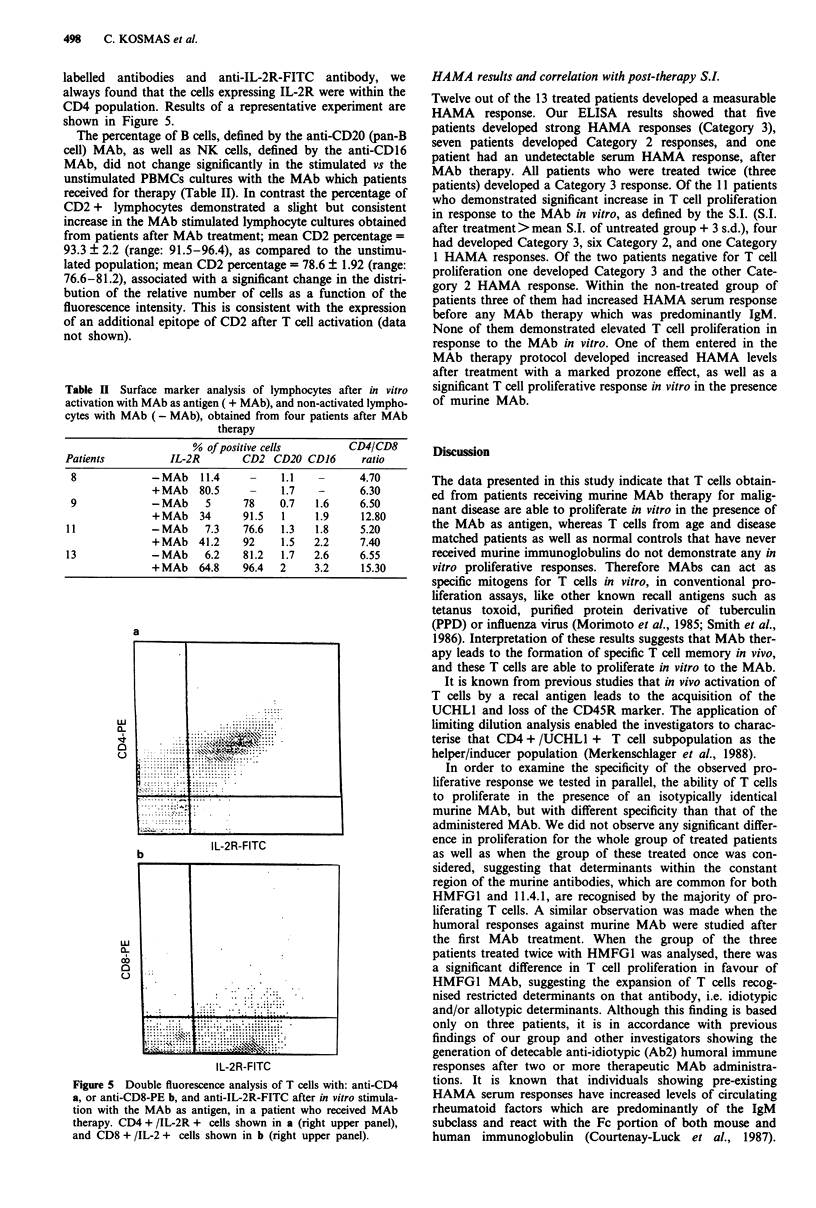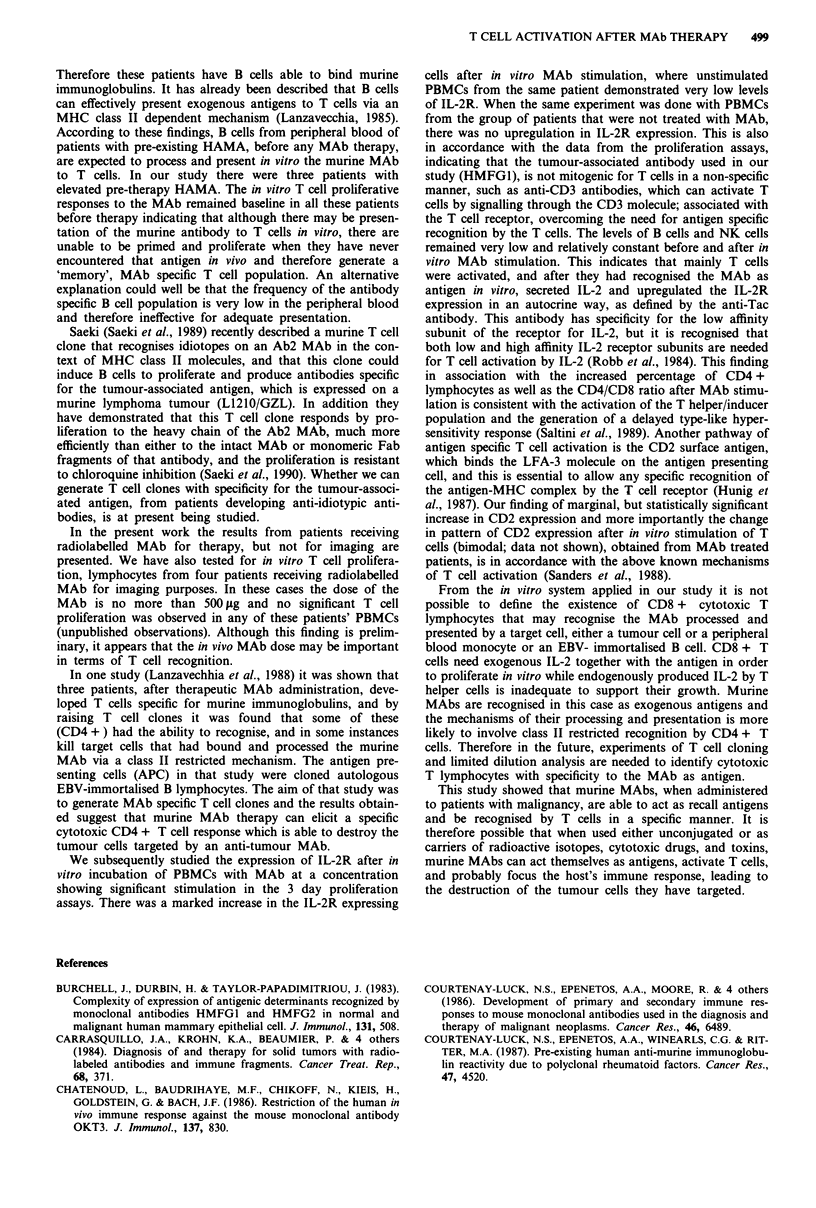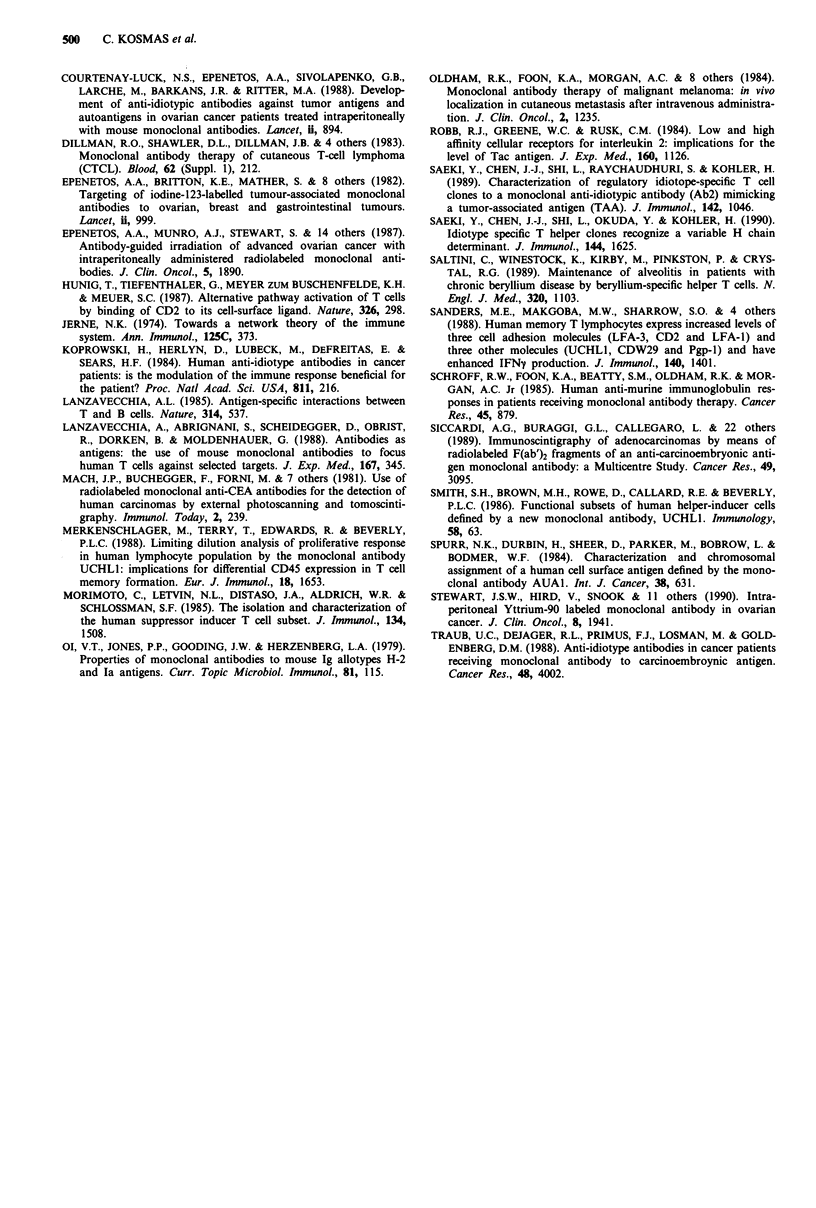# Patients receiving murine monoclonal antibody therapy for malignancy develop T cells that proliferate in vitro in response to these antibodies as antigens.

**DOI:** 10.1038/bjc.1991.337

**Published:** 1991-09

**Authors:** C. Kosmas, A. A. Epenetos, N. S. Courtenay-Luck

**Affiliations:** Imperial Cancer Research Fund Oncology Group, Royal Postgraduate Medical School, Hammersmith Hospital, London, UK.

## Abstract

Peripheral blood mononuclear cells (PBMCs) were obtained from patients receiving radioactive murine monoclonal antibody (MAb) therapy for malignant epithelial tumours, as well as normal controls, and were tested for the ability of T cells to proliferate in vitro in the presence of the MAb administered for therapy (HMFG1), and another isotypically matched antibody of irrelevant specificity (11.4.1). We studied 13 patients who had one (ten patients) or two (three patients) courses of MAB treatment, 11 age matched patients with the same histologic types of tumours, that had not received MAbs, and four normal controls. There was a consistent dose dependent in vitro T cell proliferation in 11 of the 13 patients after MAb therapy. This was not observed in the pre-therapy group of patients or normal controls, where the T cell proliferative responses remained baseline. The mean stimulation index (S.I.) in the post-therapy group was significantly higher than that of the pre-therapy patients and that of normal controls. When the in vitro T cell proliferative responses of these patients were measured in the presence of HMGF1 MAb (IgG1) and an isotypically identical, but idiotypically unrelated 11.4.1 MAb (IgG1), there was no statistically significant difference in the mean S.I. For HMFG1 vs 11.4.1 for the whole group of treated patients. When patients were separated into those who received one and those who received two MAb treatments, a significant increase in the mean S.I. was observed in the presence of HMFG1, in the group of patients receiving two treatment courses, suggesting the generation of T cells with specificity for the idiotypic component of the administered murine immunoglobulin. In order to further characterise these in vitro cellular responses we incubated PBMCs with and without an optimal concentration of the MAb (100-300 micrograms ml-1), as defined by the proliferation assay, and compared the differences in cell subpopulations. A significant increase in the percentage of cells expressing interleukin-2 receptors (IL-2R) was observed after MAb stimulation. The percentage of CD4+ lymphocytes and the CD4/CD8 ratio increased in all the cases studied, after MAb stimulation, where the percentages of B cells and NK cells remained relatively constant at less than 2-3% of the total population. We therefore conclude that murine MAbs administered to patients with cancer can lead to the generation of T cells which can recognise these MAbs as antigens when presented appropriately in vitro. The main proliferating population appears to be T helper CD4+ lymphocytes which following stimulation can release interleukin-2 leading to the expression of high levels of IL-2R.


Br. J. Cancer (1991), 64, 494–500 © Macmillan Press Ltd., 1991

### Patients receiving murine monoclonal antibody therapy for malignancy develop T cells that proliferate *in vitro* in response to these antibodies as antigens

C. Kosmas*, A.A. Epenetos & N.S. Courtenay-Luck

*Imperial Cancer Research Fund Oncology Group, and Department of Clinical Oncology, Royal Postgraduate Medical School, Hammersmith Hospital, London W12 0HS, UK.*

**Summary** Peripheral blood mononuclear cells (PBMCs) were obtained from patients receiving radioactive murine monoclonal antibody (MAb) therapy for malignant epithelial tumours, as well as normal controls, and were tested for the ability of T cells to proliferate *in vitro* in the presence of the MAb administered for therapy (HMFG1), and another isotypically matched antibody of irrelevant specificity (11.4.1).

We studied 13 patients who had one (ten patients) or two (three patients) courses of MAB treatment, 11 age matched patients with the same histologic types of tumours, that had not received MAbs, and four normal controls. There was a consistent dose dependent *in vitro* T cell proliferation in 11 of the 13 patients after MAb therapy. This was not observed in the pre-therapy group of patients or normal controls, where the T cell proliferative responses remained baseline. The mean stimulation index (S.I.) in the post-therapy group was significantly higher than that of the pre-therapy patients and that of normal controls. When the *in vitro* T cell proliferative responses of these patients were measured in the presence of HMGF1 MAb (IgG1) and an isotypically identical, but idiotypically unrelated 11.4.1 MAb (IgG1), there was no statistically significant difference in the mean S.I. For HMFG1 *vs* 11.4.1 for the whole group of treated patients. When patients were separated into those who received one and those who received two MAb treatments, a significant increase in the mean S.I. was observed in the presence of HMFG1, in the group of patients receiving two treatment courses, suggesting the generation of T cells with specificity for the idiotypic component of the administered murine immunoglobulin.

In order to further characterise these *in vitro* cellular responses we incubated PBMCs with and without an optimal concentration of the MAb (100–300 $\mu g\ ml^{-1}$), as defined by the proliferation assay, and compared the differences in cell subpopulations. A significant increase in the percentage of cells expressing interleukin-2 receptors (IL-2R) was observed after MAb stimulation. The percentage of CD4+ lymphocytes and the CD4/CD8 ratio increased in all the cases studied, after MAb stimulation, where the percentages of B cells and NK cells remained relatively constant at less than 2–3% of the total population.

We therefore conclude that murine MAbs administered to patients with cancer can lead to the generation of T cells which can recognise these MAbs as antigens when presented appropriately *in vitro*. The main proliferating population appears to be T helper CD4+ lymphocytes which following stimulation can release interleukin-2 leading to the expression of high levels of IL-2R.

Murine monoclonal antibodies (MAbs) raised against tumour-associated antigens have been used in diagnostic (Mach *et al.*, 1981; Epenetos *et al.*, 1982; Oldham *et al.*, 1984; Siccardi *et al.*, 1989) and therapeutic trials (Dillman *et al.*, 1983; Carrasquillo *et al.*, 1984; Epenetos *et al.*, 1987; Stewart *et al.*, 1990). A major limitation of murine MAbs is the development of human anti-murine immunoglobulin antibody (HAMA) responses (Schroff *et al.*, 1985; Courtenay-Luck *et al.*, 1986). It has been shown that this response contains increased levels of IgM as well as IgG antibodies. Furthermore, after repeated MAb treatment many patients develop anti-idiotypic antibodies (anti-id[1] or Ab2) (Koprowski *et al.*, 1984; Chatenoud *et al.*, 1986; Traub *et al.*, 1988). Ab2 could mimic the original tumour-associated antigen, thus providing an 'internal image', which can itself act as antigen, leading to the generation of anti-anti-idiotypic antibodies (anti-id[2] or Ab3) with binding specificities similar to the administered murine MAb (Courtenay-Luck *et al.*, 1988). The generation and maintenance of such an 'idiotypic network' is thought to be important in immune regulation, as originally postulated by Jerne (Jerne, 1974).

The humoral aspects of the human anti-mouse response have been studied in detail. What is poorly understood, is the contribution of the T cells in the generation and intregration of idiotypic networks. As previously described, when an individual is immunised with a recall antigen, such as tetanus toxoid or adenovirus, he/she will develop memory T cells able to proliferate *in vitro* in the presence of antigen, indicating successful immunisation. The secondary humoral immune response is predominantly IgG and is mediated through T and B cell interactions. The same mechanism could operate in the development of secondary humoral responses to murine immunoglobulins. In order to investigate this, we undertook a study to determine whether patients receiving therapeutic doses of murine MAbs, develop T cells able to respond *in vitro* to mitogenic signals elicited by these MAbs when appropriately presented as antigens. In addition we studied T cell subpopulations involved in this response in order to address the underlying mechanism.

### Patients, materials and methods

### *Patients and antibody protocols*

Histological confirmation of diagnosis and written informed consent from patients were obtained prior to administration of radiolabelled MAbs. Twelve patients participating in this study had stage III or IV ovarian carcinoma and one patient (male) had a malignant pleural effusion with adenocarcinoma cells, from an unknown primary (Table I). Their median age was 63 years (range: 50–68). Of the 11 patients studied before any MAb administration, seven had stage III or IV ovarian carcinoma, one (male) had malignant ascites from an adenocarcinoma of unknown primary, and three patients had

*Supported by a grant from the State Scholarship Foundation of Greece.
Correspondence: C. Kosmas.
Received 8 January 1991; and in revised form 18 April 1991.

Table I Summary of the data related to the MAb treated group of patients

| Patients | Disease and stage | Monoclonal antibody and amount injected | Isotope and radioactivity | Category of HAMA response | S.I. (±1 s.d.) in TCPA[a] |
|---|---|---|---|---|---|
| 1 | Ca Ovary Stage III | HMFG1:15 mg | 20 mCi $^{90}$Y | 3 | 3.84±0.35 |
| 2 | Ca Ovary Stage III | HMFG1:15 mg | 20 mCi $^{90}$Y | 2 | 2.58±0.33 |
| 3 | Ca Ovary Stage III | HMFG1:10 mg + HMFG2[b]:5 mg } HMFG1:20 mg | 150 mCi $^{131}$I 24 mCi $^{90}$Y | 3 | 4.97±0.90 |
| 4 | Ca Ovary Stage IV | HMFG1:15 mg — | 100 mCi $^{131}$I 50 mCi $^{90}$Y-colloid | 2 | 1.28±0.12 |
| 5 | Ad.Ca.U.P[c] | HMFG1:15 mg HMFG1:15 mg | 60 mCi $^{131}$I 60 mCi $^{131}$I | 3 | 4.24±0.53 |
| 6 | Ca Ovary Stage III | HMFG1:15 mg | 100 mCi $^{131}$I | 3 | 1.65±0.29 |
| 7 | Ca Ovary Stage III | HMFG1:15 mg HMFG1:15 mg + AUA1[d]:10 mg } | 20 mCi $^{90}$Y 100 mCi $^{131}$I | 3 | 6.45±2.23 |
| 8 | Ca Ovary Stage III | HMFG1:25 mg | 20 mCi DOTA-$^{90}$Y | 2 | 25.9±1.78 |
| 9 | Ca Ovary Stage III | HMFG1:25 mg | 20 mCi DOTA-$^{90}$Y | 2 | 7.58±1.34 |
| 10 | Ca Ovary Stage III | HMFG1:25 mg | 20 mCi DOTA-$^{90}$Y | 2 | 9.86±0.80 |
| 11 | Ca Ovary Stage III | HMFG1:25 mg | 16 mCi DOTA-$^{90}$Y | 2 | 18.66±2.67 |
| 12 | Ca Ovary Stage III | HMFG1:25 mg | 16 mCi DOTA-$^{90}$Y | 1 | 8.95±0.30 |
| 13 | Ca Ovary Stage III | HMFG1:25 mg | 16 mCi DOTA-$^{90}$Y | 2 | 14.22±1.73 |

[a]TCPA: T cell proliferation assay, S.I. in the presence of HMFG1 is shown; [b]HMFG2: MAb with similar tissue distribution to HMFG1 but more tumour specific (Burchell *et al.*, 1983); [c]Ad.Ca.U.P: Adenocarcinoma of Unknown Primary; [d]AUA1: MAb reacting with a 35 Kd antigen on a variety of epithelial neoplasms (Spurr *et al.*, 1986).

stage II or III carcinoma of the breast. Their median age was 61 years (range: 45–69). Four of the above patients were studied for *in vitro* T cell proliferation before and after therapy. Three patients (patients 3, 5, 7; see also Table I) in the study were treated twice.

Eligible patients had received their last course of cytotoxic drugs and/or radiotherapy 2 months or longer prior to MAb therapy. In the non-treated group of patients no cytotoxic drugs or radiotherapy were given for at least the last 3 months. Patients receiving steroids were not included in the study.

### *Monoclonal antibodies and mitogens*

The murine MAbs used for *in vitro* T cell proliferation assays were:

*HMFG1* This is a murine IgG1 antibody raised against human milk fat globule. It recognises a large mucin molecule. HMFG (Human Milk Fat Globule) normally expressed by the lactating breast, but also by the majority (>90%) of ovarian, breast, and other carcinomas (Burchell *et al.*, 1983).

*11.4.1* This is a murine IgG1 antibody raised against murine major histocompatibility complex antigen H-2K$^k$ (Oi *et al.*, 1979).

*Mitogens* Purified PHA (Phytohaemagglutinin; Wellcome, UK) was used as a non-specific T cell mitogen in order to exclude the possibility that the patients' T cell responses are compromised due to disseminated malignancy and/or previous cytotoxic therapy.

### *Isolation of PBMCs and tissue culture conditions*

PBMCs were isoalted from 40–50 ml of heparinised peripheral blood by centrifugation over a Lymphocyte Separation medium density gradient (Flow Lab, Irvine, UK) at 2,000 r.p.m. for 30 min. Cells recovered from the interface were washed at 1,500 r.p.m. for 10 min and at 1,100 r.p.m. for 5 min in RPMI 1640 medium (Gibco, Grand Island, NY), counted and used in proliferation assays. In some experiments, T lymphocytes were isolated by means of negative selection. Adherent cells were obtained after incubation of $3 \times 10^6$ PBMCs ml$^{-1}$ of complete tissue culture medium (TCM) [RPMI 1640 supplemented with 10% heat-inactivated foetal calf serum (FCS), 2 mmol L-glutamine, 50 U ml$^{-1}$ penicillin and 50 μg ml$^{-1}$ streptomycin] in 90 mm petri dishes (Nunc, Roskilde, Denmark), at 37°C for 2 h in a humidified atmosphere of 5% $CO_2$ and 95% air. The non-adherent cells were then depleted of B cells after incubation with magnetic beads coated with anti-CD19 MAb (DYNAL, UK), which is a pan-B cell marker. A magnetic bead to cell ratio of 75:1 was used, and after gentle mixing for 30 min at 4°C twice, the cells bound to the beads were removed by a magnetic particle concentrator (MPC, DYNAL, UK). The non-bound cells were phenotyped using fluorescein conjugated MAbs and were >95% CD3+ (T cells), <0.5% CD20+ (B cells) and <3% CD15+ (monocytes) as defined by the MAbs Leu-4, Leu-16 and Leu-M1 (Becton Dickinson, Mountain View, CA). Cell viability was >95% as determined by dye exclusion. The adherent population was then recovered by scraping the dishes with the rubber tips of sterile syringe plungers.

### *Proliferation assays*

PBMCs were incubated at $10^5$ cells per well (100 μl) of 96-well flat-bottomed microtitre plates (Sterilin, Richmond, Surrey, UK) in complete TCM by adding increasing concentrations of MAbs ($10^{-3}$–$10^3$ μg ml$^{-1}$) (HMFG1 and 11.4.1 were tested concurrently) or PHA (0.1–10 μg ml$^{-1}$). Control cultures contained cells in TCM without any MAb or PHA, in order to determine the background proliferation. In the experiments where T cells were separated from PBMCs, the adherent population (monocytes) were irradiated (3,000 rad) and used as antigen presenting cells at $5 \times 10^4$ cells per microculture well (in 50 μl volume) together with $5 \times 10^4$ T

cells (in 50 µl). The above cell populations were plated in 100 µl of TCM per well containing titrated amounts of the murine MAbs ($10^{-3}$–$10^{3}$ µg $ml^{-1}$), in a final volume of 0.2 ml. Control cultures contained only responder cells with and without MAb, only irradiated adherent cells, or responders cells (T cells) with irradiated adherent cells without MAb as antigen.

The cells were kept in culture, at 37°C in a humidified atmosphere of 5% $CO_2$ for 72 h. In the last 16 h of the total culture period they were pulsed with $^{3}$H-thymidine (0.5 µCi well) (Amersham International, UK), and harvested onto glass fibre paper using an automatic cell harvester. The incorporated radioactivity was determined using a beta-scintillation counter. Results were expressed as mean c.p.m. ± 1 s.d. of triplicate microcultures. The stimulation indices were calculated according to the formula; S.I. = c.p.m. at a given [MAb]/c.p.m. background.

### *In vitro stimulation of PBMCs with MAb and cell surface marker analysis by immunofluorescence*

PBMCs ($10^6$ cells $ml^{-1}$) from patients receiving MAb therapy and patients in the non-treated group were cultured *in vitro* in TCM with or without 100 µg $ml^{-1}$ of MAbs as in the *in vitro* T cell proliferation assays. The overall culture period was 7 days. Viable cells were separated by density gradient centrifugation, washed in PBS/0.2% BSA/0.1% sodium azide/1% heat-inactivated human AB serum and stained for 30 min at 4°C with saturating amounts of fluorescein isothiocyanate (FITC)-conjugated MAbs against: CD3 (Leu-4), CD2 (Leu-5b), CD4 (Leu-3a), CD8 (Leu-2a), for T cells, CD20 (Leu-16) for B cells, CD16 (Leu-11a) for NK cells, CD25 (Tac; low affinity IL-2R) for activated T cells, and isotype control antibodies (all from Becton-Dickinson, Mountain View, CA). For dual colour analysis cells were stained with phycoerythrin (PE)-conjugated anti-CD4, anti-CD8 antibodies and FITC-anti-CD25. After three washes the cells were resuspended in PBS and samples were analysed by an EPICS V cell sorter (Coulter Electronic, Hialeah, FL).

### *Enzyme-Linked Immunosorbent Assay (ELISA) for Human Anti-Murine Antibody (HAMA) response*

HAMA was determined by an ELISA as previously described (Courtenay-Luck *et al.*, 1986). HAMA responses were subdivided in three categories: Category 1 is a response not higher than pre-existing HAMA levels; Category 2 moderate and Category 3 strong response with a prozone phenomenon.

### *Statistical analysis*

The T cell proliferative responses of PBMCs obtained from patients before MAb therapy, normal individuals, and patients who had received MAb therapy were compared, and results expressed as the mean maximal S.I. for each group, using the two-tailed Student's *t*-test. In addition T cell proliferation in the presence of the administered, HMFG1 MAb, was compared with that of the isotype control MAb 11.4.1, both in terms of c.p.m. and S.I., by the two tailed *t*-test. *P* values of less than 0.05 were considered significant. Data are given as the mean c.p.m. or S.I. ± 1 s.d.. Treated patients were considered to be positive for *in vitro* T cell proliferation in response to the MAb, if their S.I. was greater than the mean S.I. of the untreated group of patients by 3 s.d.

### Results

### *T cell proliferation assays by $^{3}$H thymidine incorporation*

Eleven out of 13 patients receiving murine MAb therapy demonstrated an elevated T cell proliferation after *in vitro* stimulation with the MAb (HMFG1 and 11.4.1) in a dose dependent manner. The maximum proliferation was observed at concentrations of murine antibody (100–1,000 µg $ml^{-1}$) (Figures 1 and 2). None of the age matched patients with the same type of malignancy (ovarian carcinoma) or other cancers (three patients with breast carcinoma, and one with peritoneal carcinomatosis from adenocarcinoma of unknown primary) as well as normal individuals, demonstrated a dose dependent proliferation. The *in vitro* proliferative responses of the post-therapy group shown considerable variability from patient to patient and for the same patient when studied at different time intervals after therapeutic antibody administration. Significant *in vitro* responses could be observed as early as 2–4 weeks and as late as 12 months after treatment.

**Figure 1** *In vitro* proliferation of a patient's T lymphocytes; **a**, before (●), and after (○) MAb therapy, and **b**, post second therapy comparison of the T cell proliferation in the presence of HMFG1 (○) (MAb administered for the therapy) and 11.4.1 (●), an isotype control MAb.

**Figure 2** *In vitro* proliferation of a patient's T lymphocytes, who has never received MAb therapy in the presence of HMFG1 (□) and 11.4.1 (■), compared in the same experiment with the proliferative response of a patient's T cells in the presence of HMFG1 (○) and 11.4.1 (●), 4 weeks after HMFG1 MAb therapy.

### *Proliferation* in vitro *of unfractionated* vs *T cell enriched PBMCs*

As mentioned in Methods, in some experiments the T cells were separated from the whole PBMCs population by negative selection, in order to exclude the possibility that the MAb, *in vitro*, might stimulate other populations such as B lymphocytes or monocytes. Three post-therapy patients and three pre-therapy control patients were studied in parallel. There was no difference in the ability of the MAb to elicit a dose dependent proliferation when T cells plus irradiated monocytes (as feeders) or unfractionated PBMCs, were used, in all the three patients studied. Again the pre-therapy responses remained baseline whether T cells were separated or not. The background proliferation was estimated from cultures were T cells and irradiated monocytes were not pulsed with MAb. In addition, T cells alone or irradiated monocytes alone were tested for their ability to proliferate in the presence of increasing concentrations of MAb. No proliferation above the background was observed (data not shown). The results of a representative case studied before and after MAb therapy are shown in Figure 3.

### *Comparison of the Stimulation Indices (S.I.) between the groups*

Details of patients' histologic diagnosis, stage of the disease, amounts of radiolabelled MAb in mgs and radioactivity injected (in mCI of $^{131}$I or $^{90}$Y), HAMA responses and S.I. of the *in vitro* T cell proliferation assay are shown in Table I.

The mean S.I. of the group of MAb treated patients after *in vitro* stimulation with HMFG1 MAb was 8.47 ± 6.97 ($n = 13$, range: 1.28 to 25.9) which was significantly higher than the mean S.I. of the pre-therapy group, which was 1.09 ± 0.19 ($n = 11$, range: 0.90 to 1.57), $P < 0.005$, and the mean S.I. of the normal control group, which was 1.12 ± 0.18 ($n = 4$, range: 0.93 to 1.36), $P < 0.005$. There was no difference between the mean S.I. of the pre-therapy group of patients and the normal control group.

When T cells from patients receiving MAb therapy were tested for their ability to elicit an *in vitro* proliferative response in the presence of the administered MAb (HMFG1; IgG1) or an isotype control MAb (11.4.1, IgG1), studied in the same experiment, the mean S.I. for HMFG1 was 8.47 ± 6.97 and not statistically higher than the mean S.I. for 11.4.1 which was 6.44 ± 4.78, when the whole group of treated patients were examined. When patients were grouped into those treated once, and those treated twice, the following results were found. For the patients who received one MAb therapeutic course the mean S.I. for HMFG1 was 11.29 ± 7.53 ($n = 8$), which did not differ significantly from the mean S.I. for 11.4.1, which was 7.87 ± 4.90 ($n = 8$). The lymphocytes of eight out of ten patients treated once were studied for proliferation with 11.4.1 MAb. The S.I. of the two patients studied only with HMFG1 were not included in the comparative analysis, because they would obviously inbalance the comparison. For those patients treated twice the mean S.I. for HMFG1 was 5.22 ± 0.91 *vs* 2.64 ± 0.34 ($n = 3$) and the difference was statistically significant, $P < 0.01$. In addition these patients demonstrated a significantly higher proliferation in the presence of HMFG1 MAb than 11.4.1 MAb at all the concentrations of MAb *in vitro* about 10 µg ml$^{-1}$ ($P < 0.05$ by paired *t*-test). At least three experiments were performed in each case with high reproducibility of the results. Results from a representative experiment are shown (Figure 4).

**Figure 3** *In vitro* T cell proliferative responses: **a**, of unfractionated PBMCs from a patient before MAb treatment, and from another patient after HMFG1 MAb treatment, and **b**, of fractionated T cells (separated as described in Materials and methods) obtained from the same patients, described above, in the presence of HMFG1 (before □, and after O therapy) and 11.4.1 (after ● therapy).

### *Immunophenotype analysis of lymphocyte subpopulations after* in vitro *stimulation with the MAb as antigen*

When the PBMCs obtained from patients who received MAb therapy, were tested after *in vitro* stimulation with MAb we found a significant increase in the number of cells expressing IL-2R as compared with cells growing in parallel cultures without MAb. The mean percentage of IL-2R expression in unstimulated cells was 7.95 ± 2.30% (range: 5–11.4%). After *in vitro* MAb stimulation this was 55.10 ± 18.55% (range: 34–80.5%) ($P < 0.005$) in four patients who received MAb therapy (Table II). When cells from untreated patients were tested *in vitro* with or without MAb, there was no change in the number of cells expressing IL-2R, indicating the absence of stimulation by the MAb when presented as antigen.

We then estimated the CD4/CD8 ratio after *in vitro* MAb stimulation. This showed a consistent increase in the ratio in stimulated cultures; mean CD4/CD8 = 10.45 ± 3.7 (range: 6.3–15.3), when compared to unstimulated cultures from the same patient; mean CD4/CD8 = 5.7 ± 0.8 (range: 4.7–6.55), $P < 0.025$ (Table II). Again there was no change in the CD4/CD8 ratio when lymphocytes from untreated patients were cultured in the presence or absence of MAb in the culture medium (data not shown). When T cells after *in vitro* activation were phenotyped with anti-CD4 or anti-CD8-PE

**Figure 4** *In vitro* T cell proliferation, as defined by the S.I., for the three patients (Patients 5, 3, and 7; see also Table I) studied after two administrations of HMFG1 MAb, in the presence of HMFG1 and 11.4.1, at three different *in vitro* MAb concentrations (10, 100, and 1,000 µg ml$^{-1}$).

labelled antibodies and anti-IL-2R-FITC antibody, we always found that the cells expressing IL-2R were within the CD4 population. Results of a representative experiment are shown in Figure 5.

The percentage of B cells, defined by the anti-CD20 (pan-B cell) MAb, as well as NK cells, defined by the anti-CD16 MAb, did not change significantly in the stimulated *vs* the unstimulated PBMCs cultures with the MAb which patients received for therapy (Table II). In contrast the percentage of CD2 + lymphocytes demonstrated a slight but consistent increase in the MAb stimulated lymphocyte cultures obtained from patients after MAb treatment; mean CD2 percentage = 93.3 ± 2.2 (range: 91.5–96.4), as compared to the unstimulated population; mean CD2 percentage = 78.6 ± 1.92 (range: 76.6–81.2), associated with a significant change in the distribution of the relative number of cells as a function of the fluorescence intensity. This is consistent with the expression of an additional epitope of CD2 after T cell activation (data not shown).

**Table II** Surface marker analysis of lymphocytes after *in vitro* activation with MAb as antigen ( + MAb), and non-activated lymphocytes with MAb ( – MAb), obtained from four patients after MAb therapy

| Patients | | % of positive cells IL-2R | CD2 | CD20 | CD16 | CD4/CD8 ratio |
|---|---|---|---|---|---|---|
| 8 | –MAb | 11.4 | – | 1.1 | – | 4.70 |
| | +MAb | 80.5 | – | 1.7 | – | 6.30 |
| 9 | –MAb | 5 | 78 | 0.7 | 1.6 | 6.50 |
| | +MAb | 34 | 91.5 | 1 | 1.9 | 12.80 |
| 11 | –MAb | 7.3 | 76.6 | 1.3 | 1.8 | 5.20 |
| | +MAb | 41.2 | 92 | 1.5 | 2.2 | 7.40 |
| 13 | –MAb | 6.2 | 81.2 | 1.7 | 2.6 | 6.55 |
| | +MAb | 64.8 | 96.4 | 2 | 3.2 | 15.30 |

**Figure 5** Double fluorescence analysis of T cells with: anti-CD4 **a**, or anti-CD8-PE **b**, and anti-IL-2R-FITC after *in vitro* stimulation with the MAb as antigen, in a patient who received MAb therapy. CD4 + /IL-2R + cells shown in **a** (right upper panel), and CD8 + /IL-2 + cells shown in **b** (right upper panel).

*HAMA results and correlation with post-therapy S.I.*

Twelve out of the 13 treated patients developed a measurable HAMA response. Our ELISA results showed that five patients developed strong HAMA responses (Category 3), seven patients developed Category 2 responses, and one patient had an undetectable serum HAMA response, after MAb therapy. All patients who were treated twice (three patients) developed a Category 3 response. Of the 11 patients who demonstrated significant increase in T cell proliferation in response to the MAb *in vitro*, as defined by the S.I. (S.I. after treatment > mean S.I. of untreated group + 3 s.d.), four had developed Category 3, six Category 2, and one Category 1 HAMA responses. Of the two patients negative for T cell proliferation one developed Category 3 and the other Category 2 HAMA response. Within the non-treated group of patients three of them had increased HAMA serum response before any MAb therapy which was predominantly IgM. None of them demonstrated elevated T cell proliferation in response to the MAb *in vitro*. One of them entered in the MAb therapy protocol developed increased HAMA levels after treatment with a marked prozone effect, as well as a significant T cell proliferative response *in vitro* in the presence of murine MAb.

### Discussion

The data presented in this study indicate that T cells obtained from patients receiving murine MAb therapy for malignant disease are able to proliferate *in vitro* in the presence of the MAb as antigen, whereas T cells from age and disease matched patients as well as normal controls that have never received murine immunoglobulins do not demonstrate any *in vitro* proliferative responses. Therefore MAbs can act as specific mitogens for T cells *in vitro*, in conventional proliferation assays, like other known recall antigens such as tetanus toxoid, purified protein derivative of tuberculin (PPD) or influenza virus (Morimoto *et al.*, 1985; Smith *et al.*, 1986). Interpretation of these results suggests that MAb therapy leads to the formation of specific T cell memory *in vivo*, and these T cells are able to proliferate *in vitro* to the MAb.

It is known from previous studies that *in vivo* activation of T cells by a recal antigen leads to the acquisition of the UCHL1 and loss of the CD45R marker. The application of limiting dilution analysis enabled the investigators to characterise that CD4 + /UCHL1 + T cell subpopulation as the helper/inducer population (Merkenschlager *et al.*, 1988).

In order to examine the specificity of the observed proliferative response we tested in parallel, the ability of T cells to proliferate in the presence of an isotypically identical murine MAb, but with different specificity than that of the administered MAb. We did not observe any significant difference in proliferation for the whole group of treated patients as well as when the group of these treated once was considered, suggesting that determinants within the constant region of the murine antibodies, which are common for both HMFG1 and 11.4.1, are recognised by the majority of proliferating T cells. A similar observation was made when the humoral responses against murine MAb were studied after the first MAb treatment. When the group of the three patients treated twice with HMFG1 was analysed, there was a significant difference in T cell proliferation in favour of HMFG1 MAb, suggesting the expansion of T cells recognised restricted determinants on that antibody, i.e. idiotypic and/or allotypic determinants. Although this finding is based only on three patients, it is in accordance with previous findings of our group and other investigators showing the generation of detecable anti-idiotypic (Ab2) humoral immune responses after two or more therapeutic MAb administrations. It is known that individuals showing pre-existing HAMA serum responses have increased levels of circulating rheumatoid factors which are predominantly of the IgM subclass and react with the Fc portion of both mouse and human immunoglobulin (Courtenay-Luck *et al.*, 1987).

Therefore these patients have B cells able to bind murine immunoglobulins. It has already been described that B cells can effectively present exogenous antigens to T cells via an MHC class II dependent mechanism (Lanzavecchia, 1985). According to these findings, B cells from peripheral blood of patients with pre-existing HAMA, before any MAb therapy, are expected to process and present *in vitro* the murine MAb to T cells. In our study there were three patients with elevated pre-therapy HAMA. The *in vitro* T cell proliferative responses to the MAb remained baseline in all these patients before therapy indicating that although there may be presentation of the murine antibody to T cells *in vitro*, there are unable to be primed and proliferate when they have never encountered that antigen *in vivo* and therefore generate a 'memory', MAb specific T cell population. An alternative explanation could well be that the frequency of the antibody specific B cell population is very low in the peripheral blood and therefore ineffective for adequate presentation.

Saeki (Saeki *et al.*, 1989) recently described a murine T cell clone that recognises idiotopes on an Ab2 MAb in the context of MHC class II molecules, and that this clone could induce B cells to proliferate and produce antibodies specific for the tumour-associated antigen, which is expressed on a murine lymphoma tumour (L1210/GZL). In addition they have demonstrated that this T cell clone responds by proliferation to the heavy chain of the Ab2 MAb, much more efficiently than either to the intact MAb or monomeric Fab fragments of that antibody, and the proliferation is resistant to chloroquine inhibition (Saeki *et al.*, 1990). Whether we can generate T cell clones with specificity for the tumour-associated antigen, from patients developing anti-idiotypic antibodies, is at present being studied.

In the present work the results from patients receiving radiolabelled MAb for therapy, but not for imaging are presented. We have also tested for *in vitro* T cell proliferation, lymphocytes from four patients receiving radiolabelled MAb for imaging purposes. In these cases the dose of the MAb is no more than 500 μg and no significant T cell proliferation was observed in any of these patients' PBMCs (unpublished observations). Although this finding is preliminary, it appears that the *in vivo* MAb dose may be important in terms of T cell recognition.

In one study (Lanzavechhia *et al.*, 1988) it was shown that three patients, after therapeutic MAb administration, developed T cells specific for murine immunoglobulins, and by raising T cell clones it was found that some of these (CD4 + ) had the ability to recognise, and in some instances kill target cells that had bound and processed the murine MAb via a class II restricted mechanism. The antigen presenting cells (APC) in that study were cloned autologous EBV-immortalised B lymphocytes. The aim of that study was to generate MAb specific T cell clones and the results obtained suggest that murine MAb therapy can elicit a specific cytotoxic CD4 + T cell response which is able to destroy the tumour cells targeted by an anti-tumour MAb.

We subsequently studied the expression of IL-2R after *in vitro* incubation of PBMCs with MAb at a concentration showing significant stimulation in the 3 day proliferation assays. There was a marked increase in the IL-2R expressing cells after *in vitro* MAb stimulation, where unstimulated PBMCs from the same patient demonstrated very low levels of IL-2R. When the same experiment was done with PBMCs from the group of patients that were not treated with MAb, there was no upregulation in IL-2R expression. This is also in accordance with the data from the proliferation assays, indicating that the tumour-associated antibody used in our study (HMFG1), is not mitogenic for T cells in a non-specific manner, such as anti-CD3 antibodies, which can activate T cells by signalling through the CD3 molecule; associated with the T cell receptor, overcoming the need for antigen specific recognition by the T cells. The levels of B cells and NK cells remained very low and relatively constant before and after *in vitro* MAb stimulation. This indicates that mainly T cells were activated, and after they had recognised the MAb as antigen *in vitro*, secreted IL-2 and upregulated the IL-2R expression in an autocrine way, as defined by the anti-Tac antibody. This antibody has specificity for the low affinity subunit of the receptor for IL-2, but it is recognised that both low and high affinity IL-2 receptor subunits are needed for T cell activation by IL-2 (Robb *et al.*, 1984). This finding in association with the increased percentage of CD4 + lymphocytes as well as the CD4/CD8 ratio after MAb stimulation is consistent with the activation of the T helper/inducer population and the generation of a delayed type-like hypersensitivity response (Saltini *et al.*, 1989). Another pathway of antigen specific T cell activation is the CD2 surface antigen, which binds the LFA-3 molecule on the antigen presenting cell, and this is essential to allow any specific recognition of the antigen-MHC complex by the T cell receptor (Hunig *et al.*, 1987). Our finding of marginal, but statistically significant increase in CD2 expression and more importantly the change in pattern of CD2 expression after *in vitro* stimulation of T cells (bimodal; data not shown), obtained from MAb treated patients, is in accordance with the above known mechanisms of T cell activation (Sanders *et al.*, 1988).

From the *in vitro* system applied in our study it is not possible to define the existence of CD8 + cytotoxic T lymphocytes that may recognise the MAb processed and presented by a target cell, either a tumour cell or a peripheral blood monocyte or an EBV- immortalised B cell. CD8 + T cells need exogenous IL-2 together with the antigen in order to proliferate *in vitro* while endogenously produced IL-2 by T helper cells is inadequate to support their growth. Murine MAbs are recognised in this case as exogenous antigens and the mechanisms of their processing and presentation is more likely to involve class II restricted recognition by CD4 + T cells. Therefore in the future, experiments of T cell cloning and limited dilution analysis are needed to identify cytotoxic T lymphocytes with specificity to the MAb as antigen.

This study showed that murine MAbs, when administered to patients with malignancy, are able to act as recall antigens and be recognised by T cells in a specific manner. It is therefore possible that when used either unconjugated or as carriers of radioactive isotopes, cytotoxic drugs, and toxins, murine MAbs can act themselves as antigens, activate T cells, and probably focus the host's immune response, leading to the destruction of the tumour cells they have targeted.

**References**

BURCHELL, J., DURBIN, H. & TAYLOR-PAPADIMITRIOU, J. (1983). Complexity of expression of antigenic determinants recognized by monoclonal antibodies HMFG1 and HMFG2 in normal and malignant human mammary epithelial cell. *J. Immunol.*, **131**, 508.

CARRASQUILLO, J.A., KROHN, K.A., BEAUMIER, P. & 4 others (1984). Diagnosis of and therapy for solid tumors with radiolabeled antibodies and immune fragments. *Cancer Treat. Rep.*, **68**, 371.

CHATENOUD, L., BAUDRIHAYE, M.F., CHIKOFF, N., KIEIS, H., GOLDSTEIN, G. & BACH, J.F. (1986). Restriction of the human *in vivo* immune response against the mouse monoclonal antibody OKT3. *J. Immunol.*, **137**, 830.

COURTENAY-LUCK, N.S., EPENETOS, A.A., MOORE, R. & 4 others (1986). Development of primary and secondary immune responses to mouse monoclonal antibodies used in the diagnosis and therapy of malignant neoplasms. *Cancer Res.*, **46**, 6489.

COURTENAY-LUCK, N.S., EPENETOS, A.A., WINEARLS, C.G. & RITTER, M.A. (1987). Pre-existing human anti-murine immunoglobulin reactivity due to polyclonal rheumatoid factors. *Cancer Res.*, **47**, 4520.

COURTENAY-LUCK, N.S., EPENETOS, A.A., SIVOLAPENKO, G.B., LARCHE, M., BARKANS, J.R. & RITTER, M.A. (1988). Development of anti-idiotypic antibodies against tumor antigens and autoantigens in ovarian cancer patients treated intraperitoneally with mouse monoclonal antibodies. *Lancet*, **ii**, 894.

DILLMAN, R.O., SHAWLER, D.L., DILLMAN, J.B. & 4 others (1983). Monoclonal antibody therapy of cutaneous T-cell lymphoma (CTCL). *Blood*, **62** (Suppl. 1), 212.

EPENETOS, A.A., BRITTON, K.E., MATHER, S. & 8 others (1982). Targeting of iodine-123-labelled tumour-associated monoclonal antibodies to ovarian, breast and gastrointestinal tumours. *Lancet*, **ii**, 999.

EPENETOS, A.A., MUNRO, A.J., STEWART, S. & 14 others (1987). Antibody-guided irradiation of advanced ovarian cancer with intraperitoneally administered radiolabeled monoclonal antibodies. *J. Clin. Oncol.*, **5**, 1890.

HUNIG, T., TIEFENTHALER, G., MEYER ZUM BUSCHENFELDE, K.H. & MEUER, S.C. (1987). Alternative pathway activation of T cells by binding of CD2 to its cell-surface ligand. *Nature*, **326**, 298.

JERNE, N.K. (1974). Towards a network theory of the immune system. *Ann. Immunol.*, **125C**, 373.

KOPROWSKI, H., HERLYN, D., LUBECK, M., DEFREITAS, E. & SEARS, H.F. (1984). Human anti-idiotype antibodies in cancer patients: is the modulation of the immune response beneficial for the patient? *Proc. Natl Acad. Sci. USA*, **811**, 216.

LANZAVECCHIA, A.L. (1985). Antigen-specific interactions between T and B cells. *Nature*, **314**, 537.

LANZAVECCHIA, A., ABRIGNANI, S., SCHEIDEGGER, D., OBRIST, R., DORKEN, B. & MOLDENHAUER, G. (1988). Antibodies as antigens: the use of mouse monoclonal antibodies to focus human T cells against selected targets. *J. Exp. Med.*, **167**, 345.

MACH, J.P., BUCHEGGER, F., FORNI, M. & 7 others (1981). Use of radiolabeled monoclonal anti-CEA antibodies for the detection of human carcinomas by external photoscanning and tomoscintigraphy. *Immunol. Today*, **2**, 239.

MERKENSCHLAGER, M., TERRY, T., EDWARDS, R. & BEVERLY, P.L.C. (1988). Limiting dilution analysis of proliferative response in human lymphocyte population by the monoclonal antibody UCHL1: implications for differential CD45 expression in T cell memory formation. *Eur. J. Immunol.*, **18**, 1653.

MORIMOTO, C., LETVIN, N.L., DISTASO, J.A., ALDRICH, W.R. & SCHLOSSMAN, S.F. (1985). The isolation and characterization of the human suppressor inducer T cell subset. *J. Immunol.*, **134**, 1508.

OI, V.T., JONES, P.P., GOODING, J.W. & HERZENBERG, L.A. (1979). Properties of monoclonal antibodies to mouse Ig allotypes H-2 and Ia antigens. *Curr. Topic Microbiol. Immunol.*, **81**, 115.

OLDHAM, R.K., FOON, K.A., MORGAN, A.C. & 8 others (1984). Monoclonal antibody therapy of malignant melanoma: *in vivo* localization in cutaneous metastasis after intravenous administration. *J. Clin. Oncol.*, **2**, 1235.

ROBB, R.J., GREENE, W.C. & RUSK, C.M. (1984). Low and high affinity cellular receptors for interleukin 2: implications for the level of Tac antigen. *J. Exp. Med.*, **160**, 1126.

SAEKI, Y., CHEN, J.-J., SHI, L., RAYCHAUDHURI, S. & KOHLER, H. (1989). Characterization of regulatory idiotope-specific T cell clones to a monoclonal anti-idiotypic antibody (Ab2) mimicking a tumor-associated antigen (TAA). *J. Immunol.*, **142**, 1046.

SAEKI, Y., CHEN, J.-J., SHI, L., OKUDA, Y. & KOHLER, H. (1990). Idiotype specific T helper clones recognize a variable H chain determinant. *J. Immunol.*, **144**, 1625.

SALTINI, C., WINESTOCK, K., KIRBY, M., PINKSTON, P. & CRYSTAL, R.G. (1989). Maintenance of alveolitis in patients with chronic beryllium disease by beryllium-specific helper T cells. *N. Engl. J. Med.*, **320**, 1103.

SANDERS, M.E., MAKGOBA, M.W., SHARROW, S.O. & 4 others (1988). Human memory T lymphocytes express increased levels of three cell adhesion molecules (LFA-3, CD2 and LFA-1) and three other molecules (UCHL1, CDW29 and Pgp-1) and have enhanced IFN$\gamma$ production. *J. Immunol.*, **140**, 1401.

SCHROFF, R.W., FOON, K.A., BEATTY, S.M., OLDHAM, R.K. & MORGAN, A.C. Jr (1985). Human anti-murine immunoglobulin responses in patients receiving monoclonal antibody therapy. *Cancer Res.*, **45**, 879.

SICCARDI, A.G., BURAGGI, G.L., CALLEGARO, L. & 22 others (1989). Immunoscintigraphy of adenocarcinomas by means of radiolabeled $F(ab')_2$ fragments of an anti-carcinoembryonic antigen monoclonal antibody: a Multicentre Study. *Cancer Res.*, **49**, 3095.

SMITH, S.H., BROWN, M.H., ROWE, D., CALLARD, R.E. & BEVERLY, P.L.C. (1986). Functional subsets of human helper-inducer cells defined by a new monoclonal antibody, UCHL1. *Immunology*, **58**, 63.

SPURR, N.K., DURBIN, H., SHEER, D., PARKER, M., BOBROW, L. & BODMER, W.F. (1984). Characterization and chromosomal assignment of a human cell surface antigen defined by the monoclonal antibody AUA1. *Int. J. Cancer*, **38**, 631.

STEWART, J.S.W., HIRD, V., SNOOK & 11 others (1990). Intraperitoneal Yttrium-90 labeled monoclonal antibody in ovarian cancer. *J. Clin. Oncol.*, **8**, 1941.

TRAUB, U.C., DEJAGER, R.L., PRIMUS, F.J., LOSMAN, M. & GOLDENBERG, D.M. (1988). Anti-idiotype antibodies in cancer patients receiving monoclonal antibody to carcinoembroynic antigen. *Cancer Res.*, **48**, 4002.